# Divergent Effects of Liraglutide, Exendin-4, and Sitagliptin on Beta-Cell Mass and Indicators of Pancreatitis in a Mouse Model of Hyperglycaemia

**DOI:** 10.1371/journal.pone.0104873

**Published:** 2014-08-13

**Authors:** Angeles Mondragon, Daniel Davidsson, Styliana Kyriakoudi, Annika Bertling, Rosa Gomes-Faria, Patrizia Cohen, Stephen Rothery, Pauline Chabosseau, Guy A. Rutter, Gabriela da Silva Xavier

**Affiliations:** 1 Section of Cell Biology, Division of Diabetes, Endocrinology and Metabolism, Department of Medicine, Faculty of Medicine, Imperial College London, London, United Kingdom; 2 Imperial College Healthcare NHS Trust, Department of Pathology, St. Mary's Hospital, London, United Kingdom; 3 FILM, Imperial College London, London, United Kingdom; University of Lancaster, United Kingdom

## Abstract

**Aims:**

Glucagon-like peptide-1 (GLP-1) receptor agonists and dipeptidyl peptidase-4 (DPP4) inhibitors improve glucose tolerance by still incompletely understood mechanisms. Each class of antihyperglycemic drugs has also been proposed to increase pancreatitis risk. Here, we compare systematically the effects of two widely-used GLP-1 analogues, liraglutide and exendin-4, and the DPP4 inhibitor, sitagliptin, in the mouse.

**Methods:**

C57BL6 mice were maintained for 131 days on a normal diet (ND) or a diet comprising 60% fat (HFD) before measurements of fasting blood glucose and insulin, and intraperitoneal glucose tolerance. Beta- and alpha- cell volume, and Reg3b immunoreactivity, were measured by immunohistochemical analysis of pancreatic slices.

**Results:**

Whereas liraglutide (200 µg/kg) and exendin-4 (10 µg/kg) treatment reduced body weight and/or improved glucose tolerance, sitagliptin (10 mg/kg) was without effect on either parameter. Liraglutide caused a sharp reduction in beta-cell mass in both ND and HFD mice, whereas exendin-4 exerted no effect. By contrast, sitagliptin unmasked an action of high fat diet to increase beta-cell mass. Reg3B positive area was augmented by all three agents in normal chow-fed mice, whilst sitagliptin and exendin-4, but not liraglutide, affected this parameter in HFD animals. Correspondingly sitagliptin, but not the GLP-1 analogues, increased circulating amylase levels in ND and HFD mice.

**Conclusions:**

Liraglutide improves glucose tolerance in the mouse whilst exerting relatively modest effects on pancreatitis risk. Conversely, exendin-4 and sitagliptin, at doses which exert, respectively, minor or no effects on metabolic parameters, lead to signs of pancreatitis.

## Introduction

The incretin, glucagon-like peptide 1 (GLP-1), secreted after meal ingestion from L-cells found primarily in the distal intestine [1], promotes the secretion of insulin and somatostatin by pancreatic beta- and delta- cells, respectively, and decreases glucagon production from alpha-cells, as well as appetite and gastric emptying Together with a suggested action on beta-cell proliferation [2], and an absence of adverse effects such as weight gain and hypoglycaemic episodes [3], GLP-1 is an ideal candidate as a drug to treat type 2 diabetes (T2D)[4]. However, GLP-1 has a short circulating half-life (<2 min) due to its rapid degradation by dipeptidyl peptidase 4 (DPP-4), such that its therapeutic use requires continuous administration or the engineering of GLP-1 mimetics with longer circulating life-times [5]. Consequently the last decade has seen the development of two classes of GLP-1-based drugs for T2D: GLP-1 receptor (GLP-1R) agonists that mimic GLP-1 but are resistant to degradation by DPP-4, and DPP-4 inhibitors.

Although an efficient drug class for the treatment of T2D [6], recent data indicate that long-term administration of the GLP-1R agonists and DPP4 inhibitors may be linked to an increased risk of pancreatitis and pancreatic cancer [7]–[11]. The risk of pancreatic cancer conferred by the usage of these anti-diabetic drugs is difficult to assess as patients with a history of pancreatitis and diabetes are in any case at increased risk of developing pancreatic cancer [12], [13]. Three commonly used agents are exenatide/exendin-4 and liraglutide, which are GLP-1 mimetics, and sitagliptin, a DPP4 inhibitor.

The U.S. FDA issued a warning to healthcare professionals about the possible increased risk of pancreatitis in T2D patients taking sitagliptin following 88 cases of acute pancreatitis related with sitagliptin use in 2009; pancreatitis was also associated with exenatide use [14], [15]. Subsequently, liraglutide, exendin-4 and sitagliptin have all been variously associated with pancreatitis risk in patients and rodent models [10], [16]–[28] [29]. These results raised concerns as chronic pancreatitis has been shown to increase the risk of pancreatic cancer [26]. There is, nonetheless, a lack of information from human pancreata; data from patients on long-term treatment are not currently available, and the use of the AERS to assess drug safety is arguably imperfect [9]. The evidence for an association between GLP-1-based therapy and the development of pancreatitis is intensified by the fact that all of the developed agents which have been on the market long enough have now been linked to cases of pancreatitis [17].

Although much has been published on the potential risk of pancreatitis and pancreatic cancer from administration of GLP-1 mimetics, there have equally been studies which demonstrate no effects on this parameter [30]–[38]. A recent study by Ellenbroek and colleagues [39] demonstrated that mice administered liraglutide prior to the start of a six-week long high fat diet regimen remained normoglycaemic and exhibited decreased beta cell mass, possibly due to improved insulin sensitivity. In total liraglutide's efficacy and safety have been investigated in more than 5000 patients through 20 clinical trials; in a number of these studies markers of beta-cell function were also analysed leading to the indication of improved beta-cell function [40], [41], amongst other potential beneficial effects [41]–[43]. The FDA and EMA exert panels have also recently ruled that available data do not confirm recent concerns over an increased risk for pancreatic side effects with GLP-1-based diabetes therapies.

The aims of the present study were, therefore, to probe in a mouse model of diet-induced glucose intolerance [44]–[46] the propensity of the GLP-1 receptor agonists- liraglutide and exendin-4- and the DPP4 inhibitor-sitagliptin- to cause signs of pancreatitis, whilst comparing the action of each on weight gain, glucose homeostasis and beta-cell mass.

## Methods

### Materials

All general chemicals and tissue culture reagents were purchased from Sigma (Dorset, U.K.) or Invitrogen (Paisley, U.K.), unless otherwise stated.

### Animals

All *in vivo* procedures were approved by the U.K. Home Office according to the Animals (Scientific Procedures) Act 1986 and were performed at the Central Biomedical Service, Imperial College, London, U.K. C57BL/6 male mice were purchased from Charles River (U.K.). For high fat diet treatment, mice were placed on a high fat diet at eight weeks of age for eight weeks (60% [wt/wt] fat content; Research Diet, New Brunswick, NJ, USA). Mice were housed at two to five animals per cage in a pathogen-free facility with a 12-hour light-dark cycle. Animals were fed *ad libitum* with a standard mouse chow diet (Research Diet, New Brunswick, NJ) unless otherwise stated. Mice were culled by cervical dislocation.

### Intraperitoneal glucose tolerance test

Mice were fasted for 16 h, with water available *ad libitum* prior to the test. Glucose tolerance tests were conducted at 09:00 on each experimental day. Glucose tolerance was assessed by intraperitoneal administration of glucose (1 g/kg).

### Administration of GLP-1 mimetics

C57BL/6 mice (eight weeks old) were maintained on a normal chow or high fat (60%; Lillico) diet for eight weeks prior to the start of the injection regime. At 16 weeks of age, mice were injected daily with saline, liraglutide (200 µg/kg [47]; Bachem, Bubendorf, Switzerland), exendin-4 (10 µg/kg [27]; Polypeptide Group SCI537 Strasbourg, France), or sitagliptin (10 mg/kg [48]; Sigma) at the start of the dark cycle.

### Immunohistochemistry and widefield microscopy

Isolated pancreases were fixed in 10% buffered formalin and embedded in paraffin wax within 24 h of removal. Head-to-tail sections (5 µm lengthwise) were cut and incubated overnight at 37°C on superfrost slides. Slides were submerged sequentially in Histoclear (Sigma) followed by decreasing concentrations of industrial methylated spirits for removal of paraffin wax. Permeabilised slices were blotted with primary antibodies against insulin (DAKO, Cambridgeshire, U.K.), glucagon (Sigma) and Reg3B (R&D Systems, Abingdon, U.K.) with antigen unmasking using vector antigen unmasking solution (Vector Laboratories, Peterborough, U.K.), and visualised with Alexa Fluor 488 or 568 secondary antibodies (Invitrogen). Specimens were mounted on glass slides using Vectashield hard set with DAPI (Vector Laboratories).

Images were captured on a Zeiss Axio Observer.Z1 Motorised Inverted Widefield Microscope fitted with a Hamamatsu Flash 4.0 Camera using Plan-Apochromat 20×/0.8 M27 air objective with Colibri.2 LED illumination. Data acquisition was controlled by Zeiss Zen Blue 2012 software configured at a bit depth of 16-bit and binning mode 2×2.

Whole tissue tiled preview scans were obtained using an EC Plan-Neofluar 10×/0.3 Ph1 air objective with phase contrast. For Reg3B analysis, a 20 point array was chosen at random with point focusing achieved with the Plan-Apochromat 20×/0.8 M27 air objective using EGFP 488 filter as the reference channel for local surface focusing.

Fluorescence quantification was achieved using Fiji [49]. Threshold measurements were taken for total tissue area using particle size 200-infinity and fluorescence area using particle size 20-infinity for each of the 20 point arrays. Ratios were subsequently calculated with an *in-house* macro for comparative analyses between sample groups. We verified that our macro was providing a measure of change in fluorescence through comparison with manual scoring of the slides using an Nikon TS100-F microscope fitted with a LED light source and appropriate filters (Fig. S1; Nikon, London, U.K.). All image capture and analysis were performed with the operator blinded to the identity of the slides.

### Measurement of plasma amylase, lipase and GLP-1

Blood (200 µl) was removed by cardiac puncture from mice killed by cervical dislocation. Plasma was collected using high speed (2000 *g*, 5 min at 4°C) centrifugation in heparin-coated Microvette tubes containing EDTA (Sarstedt, Leicester, U.K.). Plasma amylase and lipase levels were assessed using the lipase and amylase assay kits from Abcam (Cambridge, U.K.). Plasma GLP-1 levels were assessed as previously described [45].

### Histopathology

Slices from pancreases prepared as detailed under ‘*Immunohistochemistry and widefield microscopy’* were stained with eosin and haemotoxylin and subjected to histopathological analysis. Qualitative analysis of the following parameters were performed- endocrine islet size variation and inflammation; cytoplasmic vacuole, nucleus size variation, necrolysis/autolysis, autolysis/fibrosis, lobular inflammation, septal inflammation in exocrine pancreas; peripancreatic fat inflammation.

### Statistical analysis

Data are the means ± S.E. for the number of observations indicated. Statistical significance and differences between means were assessed by Student's t-test with Bonferroni correction for multiple analyses as appropriate in Excel (Microsoft).

## Results

### Liraglutide and exendin-4 restrict weight gain, but liraglutide is more effective at maintaining glucose tolerance in high fat diet-fed mice

**Figure 1:**
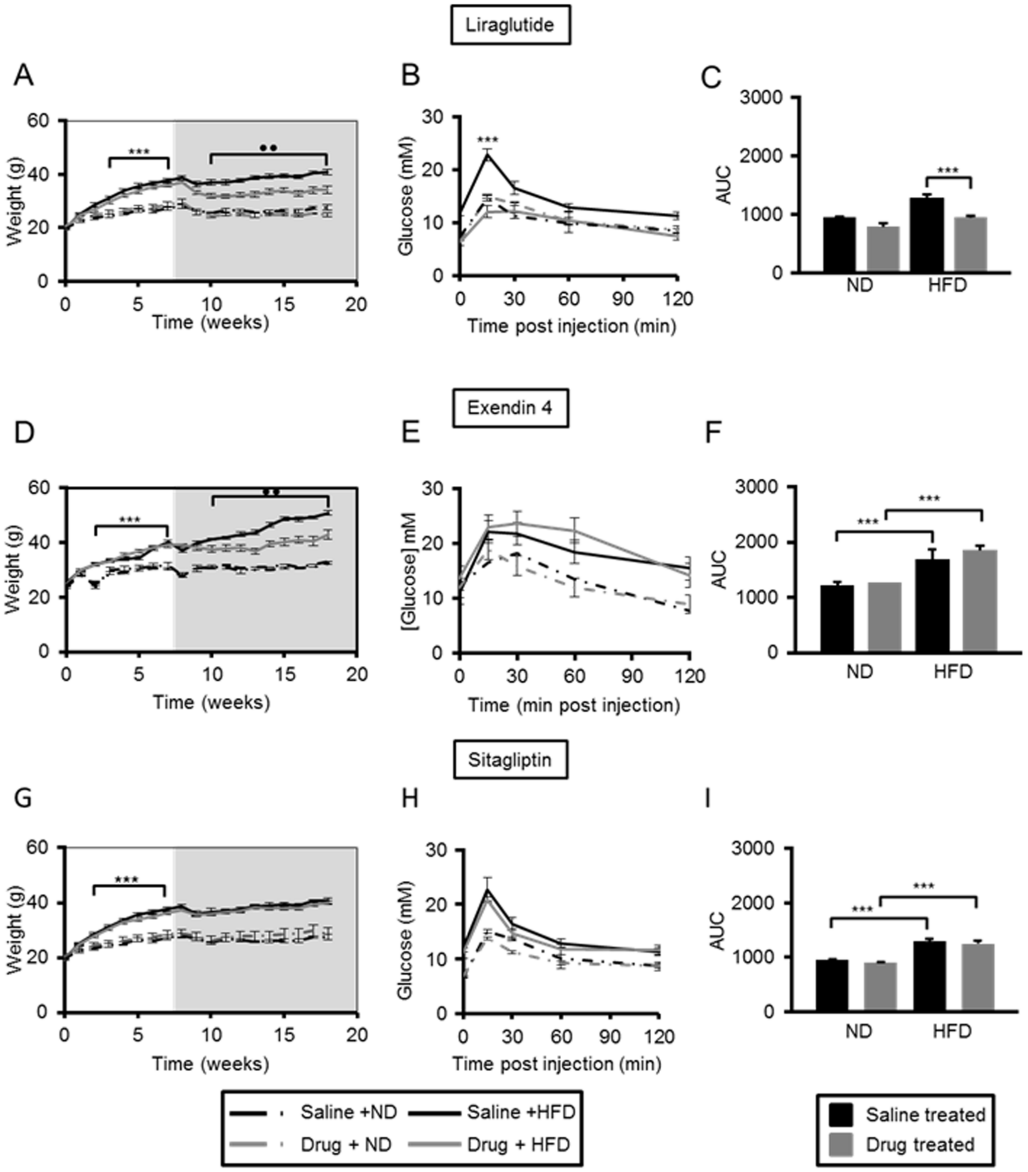
Liraglutide and exendin-4 are effective weight management drugs in high fat-fed C57BL/6 mice, but liraglutide is more effective at improving glucose tolerance. The weight of male C57BL/6 mice was monitored on a weekly basis over a period of eight weeks (white area) on high fat (HFD) or normal chow diet (ND), followed by 75 days (grey area) of daily intraperitoneal injections of saline, or a GLP-1 mimetic- liraglutide (panels A–C), exendin-4 (panels D–F), or sitagliptin (panels G–I). Intra-peritoneal glucose tolerance tests were performed after 75 days of treatment (B–C, E–F, H–I). Time courses (B, E, H) and the corresponding areas under the curve (C, F, I) are shown. p≤0.001, ***; p≤0.01, **, n = 3–7 mice.

Administration of a high fat (60% fat) diet (HFD) led to marked weight gain in mice as expected (Fig. 1A, B, C;). Correspondingly, glucose tolerance was impaired, with an increase in the area under the curve (AUC) of 56.8±4.9% (p≤0.001, *n* = 16 per group) *vs* mice maintained on a normal chow diet (Fig. S2). We chose doses and administration routes of liraglutide, exendin-4 and sitagliptin that were previously shown to be effective in mice [27], [47], [48]. Neither liraglutide, exendin-4, nor sitagliptin exerted any effect on weight gain or glucose tolerance in mice maintained on a normal chow diet (Fig. 1). Administration of liraglutide and exendin-4 led to a 23.9±3.0% (p≤0.01, *n* = 4 per group) and a 37.4±5.0% (p≤0.01, *n* = 3 per group), decrease in weight gain in mice on a high fat diet over the course of the drug regimen, respectively (Fig. 1A, B). Administration of liraglutide led to an improvement in glucose tolerance in HFD mice at the end of the 75 days' treatment period (26.3±2.1% decrease in AUC *vs* mice high fat diet that had been administered saline, p≤0.01), such that glucose tolerance was indistinguishable from that observed in mice maintained on a normal diet (Fig. 1D, G). In our hands, neither exendin-4 nor sitagliptin improved glucose tolerance in mice maintained on HFD by the end of the treatment period (Fig. 1E–F, H–I).

### Long term adminstration of liraglutide, exendin-4 and sitagliptin exert differing effects on beta-cell mass but no effect on alpha-cell mass

**Figure 2:**
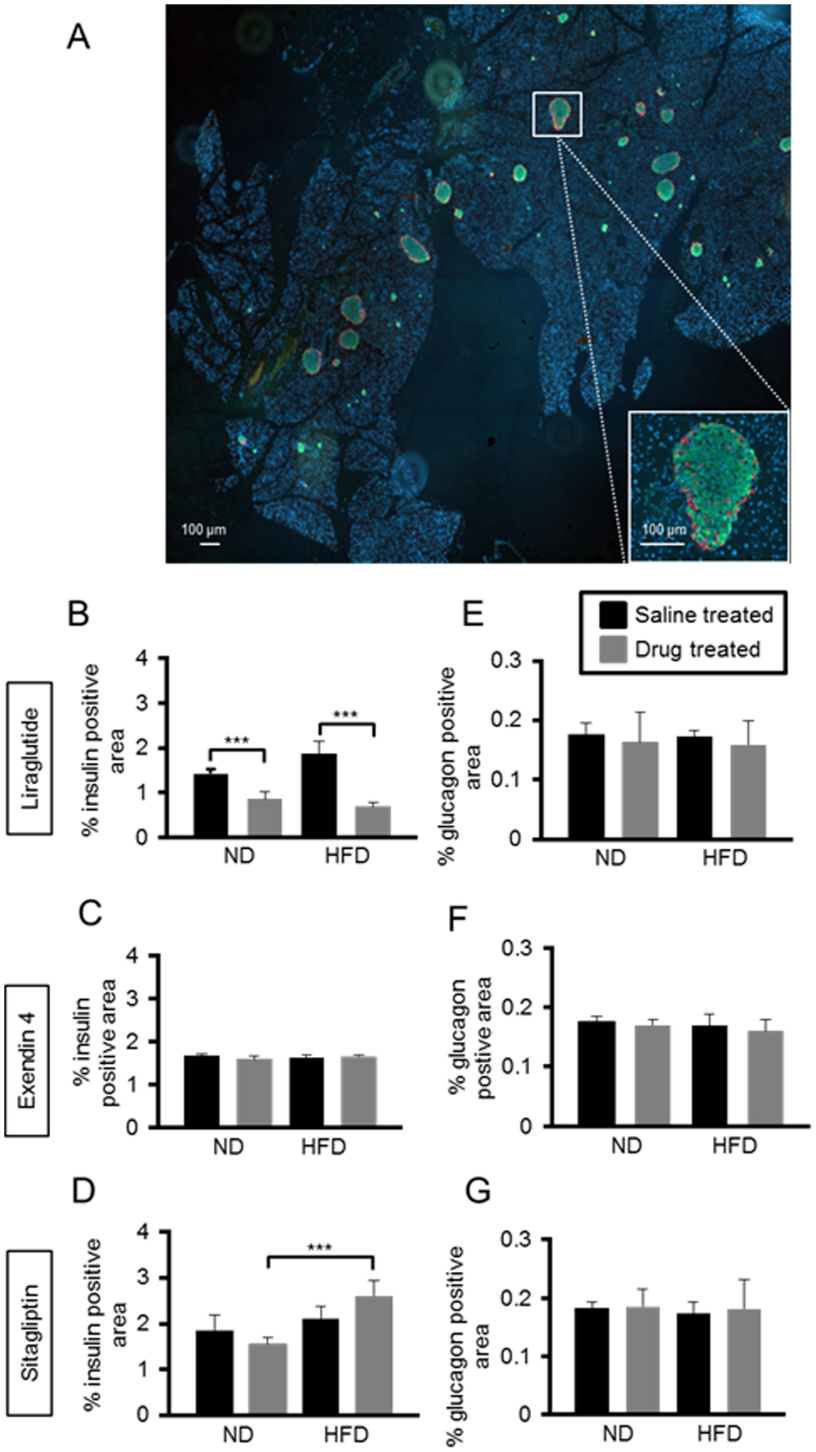
Long term adminstration of exendin-4, liraglutide and sitagliptin exerts different effects on beta-cell mass but no effect on alpha-cell mass. Representative images of pancreatic sections stained for insulin (green), glucagon (red) and nuclei (DAPI, blue) (A). Pancreatic sections from mice treated with liraglutide (B, E), exendin-4 (C, F) and sitagliptin (D, G) were examined. Beta-cell mass (B, C, D), and alpha-cell mass (E, F, G) were measured from whole section scans as described in ‘Materials and Methods’. p≤0.001, ***; p≤0.01, n = 3–7 mice.

Pancreatic beta- and alpha-cell mass (Fig. 2A) were quantified in pancreatic slices as described under Materials and Methods. Administration of liraglutide markedly lowered beta-cell mass in mice maintained on a normal chow diet (39±9.8%, p≤0.01, *n* = 3 per group) or high fat diet (62.2±4.5%, p≤0.001, *n* = 4 per group) *vs* control mice (Fig. 2B).Administration of exendin 4 had effect on beta cell mass (Fig. 2C). Interestingly, whilst a HFD alone exerted no effects on beta-cell mass in the absence of drug treatment, administration of sitagliptin led to the unmasking of an action of HFD to increase beta cell mass (40.6%, p≤0.01, *n* = 4 per group), when compared with mice treated with the drug on a normal chow diet (Fig. 2D). No observable differences in alpha-cell mass were found between any of the cohorts (Fig. 2E–G).

### Long term administration of liraglutide, exendin-4 and sitagliptin lead to increased Reg3b immunoreactivity

**Figure 3:**
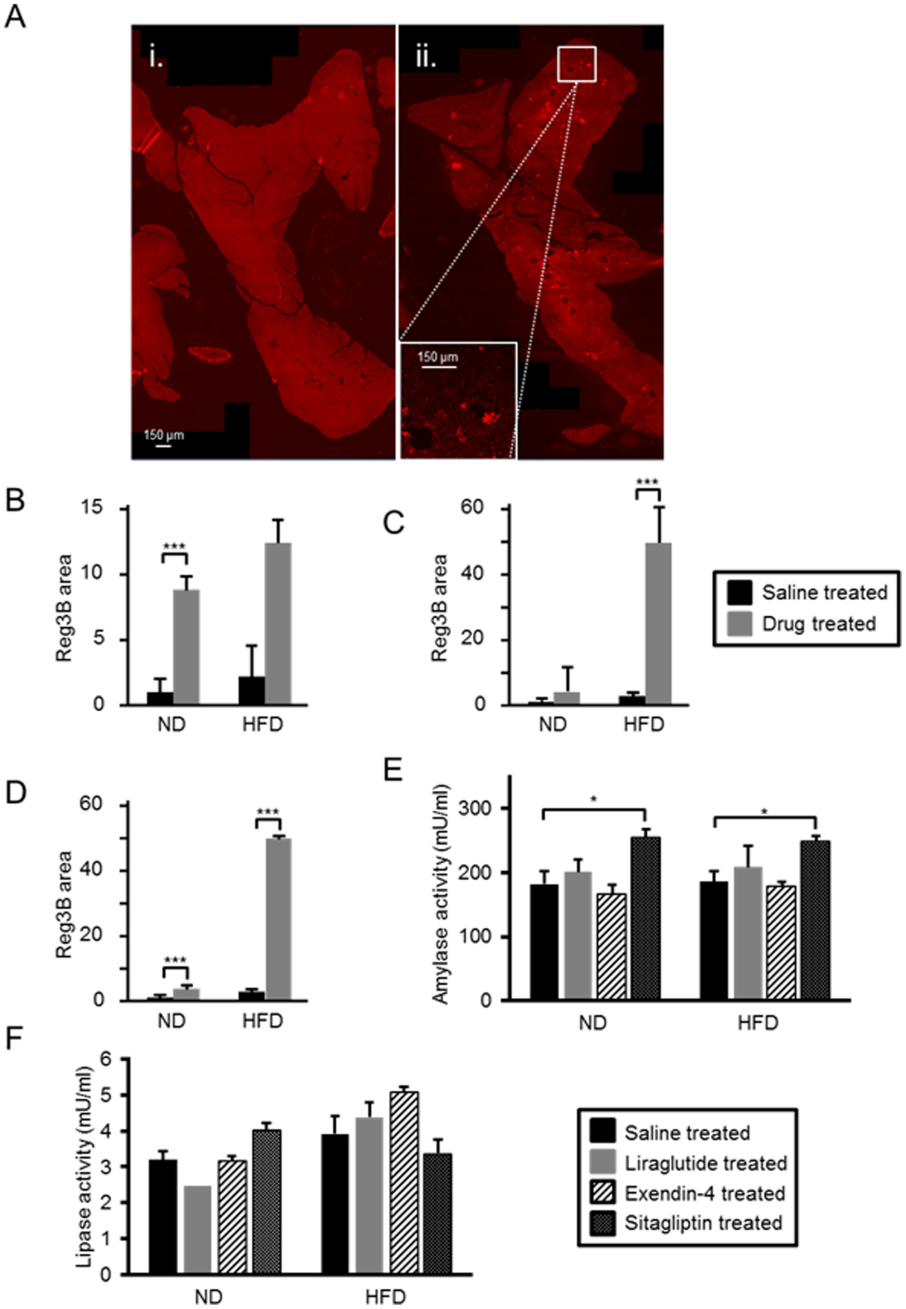
C57BL/6 mice do not display clinical signs of pancreatitis following 75 days' treatment with liraglutide, exendin-4, or sitagliptin. Reg3b immunoreactivity was assessed in pancreatic sections from mice treated with saline (Ai), liraglutide (B), exendin-4 (Aii, C) and sitagliptin (D), and normalised to Reg3b area observed in pancreata from saline treated mice, as described in ‘Materials and Methods’. Plasma amylase (E) and lipase (F) levels were measured after 75 days treatment with saline, liraglutide, exendin-4 or sitagliptin. ND, normal chow diet; HFD, high fat diet. p≤0.05, *; p≤0.01, **, n = 3–7 mice.

Administration of liraglutide, exendin-4 and sitagliptin led to increased Reg3b immunoreactivity to differing extents in mouse pancreata, and depending on diet (Fig. 3A–D). Thus, the increase in Reg3b signal area associated with administration of liraglutide to mice on a normal diet was 3.3±0.6- fold (p≤0.001, *n* = 3 per group) (Fig. 3B). Interestingly, administration of liraglutide did not exacerbate the effects of high fat diet alone (Fig. 3B). Administration of exendin-4, on the other hand, led to a signal increase of 5.8±0.8-fold (p≤0.001, *n* = 4 per group) in the Reg3b signal in mice on high fat diet (Fig. 3C), as well as in ND animals. Administration of sitagliptin to mice on normal diet resulted in a 3.9±0.9-fold increase in Reg3b signal (p≤0.05, *n* = 3 per group), and this increase was of 18.6±6.6-fold (p≤0.01, *n* = 4 per group) in mice on high fat diet (Fig. 3D).

### Clinical measures of pancreatitis

There were no statistically significant changes in plasma amylase activity in mice that were administered liraglutide or exendin-4 *vs* mice administered saline (Fig. 3E). However, administration of sitagliptin to animals on normal diet led to a 1.4-fold increase in amylase activity (p≤0.01, *n* = 3 per group) and a 1.3- fold increase in mice on a high fat diet (p≤0.01, *n* = 4 per group) (Fig. 3E).

There were no detectable differences in plasma lipase activity in mice on a normal chow diet administered any of the three drugs when compared to animals administered saline (Fig. 3F). Likewise, there was no significant change in plasma lipase activity in mice that were administered saline on a high fat diet *vs* normal diet (Fig. 3F). Furthermore, administration of liraglutide and exendin-4 in combination with a high fat diet also failed to affect plasma lipase activity. We observed no detectable changes in plasma lipase activity in animals maintained on a normal chow diet and administered any of the three drugs when compared to animals administered saline (Fig. 3F).

Histopathological analysis revealed no significant differences in pathological status of pancreatic slices from the different treatment groups, with a potential indication of an increase in inflammatory response in mice subjected to a high fat diet *vs* mice that have been maintained on a normal chow diet (Fig. S3).

## Discussion

Animal studies do not necessarily predict with certainty what will happen in humans during similar treatment protocols [50]. Nonetheless, given the poor prognosis for patients diagnosed with pancreatic cancer, and the lack of risk data from long term studies of patients on these treatments, there is a need to examine the risk of pancreatitis following long-term treatment with GLP-1 receptor agonists in model systems. To circumvent some of the potential problems involved with studying human disease risk in mouse models, we have selected a model which reflects at least several key elements of T2D in humans [44]. Importantly, we have administered drugs to mice that already exhibit glucose dyshomeostasis, in contrast to a recent study [39] whereby liraglutide was administered prior to the start of the high fat diet regimen.

Of the three agents tested, liraglutide, at the dose tested here, was overall the most effective at decreasing weight gain and improving glucose tolerance by the end of the 75 days' treatment (Fig. 1). Interestingly, this change was accompanied by a *decrease* in beta-cell mass in mice on both normal chow diet and high fat diet (Fig. 2B). These data are consistent with a recent report showing that mice administered liraglutide and maintained on a high fat diet for six weeks, exhibited decreased beta-cell mass possibly due to improved insulin sensitivity [39]. Interestingly, mice on a high fat diet in the earlier study by Ellenbroek and colleagues [39] remained normoglycaemic throughout the study, since they were given liraglutide prior to starting the high fat diet regime. More closely mimicking the use of the drug in man, our mice were rendered hyperglycaemic prior to drug administration and the drug regimen was longer than that reported in [39]. Suggesting these differences in protocol may be important [39], we saw no changes in alpha-cell mass during liraglutide treatment in the present studies. By contrast, neither exendin-4 nor sitagliptin exerted any effect on beta-cell mass in the present study, albeit under conditions here where neither drug exerted significant effects on glucose homeostasis.

In this study we administered sitagliptin by intraperitoneal injection as daily oral gavages were impractical and would mean increasing the number of mice we needed to use per cohort, with the associated ethical issues. Administration by admixture was prohibitively costly. To assess the efficacy of adminstration of sitagliptin by intraperitoneal injection, we measured plasma GLP-1 levels one hour after co-injection of glucose (1 g/kg) and sitagliptin (10 mg/kg), and demonstrated that this was effective at raising plasma GLP-1 content, *vs* saline control (Fig. S1C).

An important prompt for the present investigation was the controversy that exists over whether long term administration of GLP-1 mimetics may lead to pancreatitis and pancreatic cancer. One confounding factor may be the propensity for rodent models to develop spontaneous pancreatic lesions [51]. Our observations indicate that rodents may indeed manifest signs of spontaneous pancreatic lesions but that some of the signals for pancreatic disease are exaggerated by exposure to the GLP-1 mimetics and DDP 4 inhibitor drug class (Fig. 3, Fig. S1). We have also observed that prolonged exposure to high fat diet has a tendency to lead to increased Reg3B immunoreactivity (Fig. 3B–D) even though there were no signs of overt clinical pancreatitis. Clinical pancreatitis is indicated by increases in the activity of both plasma amylase and lipase. Although we saw increased pancreatic content of Reg3B, a marker of pancreatitis, by immunohistochemical analysis, with all three GLP-1 mimetics used in this study (Fig.3A–D), we did not observe increases in plasma amylase or lipase with administration of liraglutide and exendin-4 in conjunction with the high fat diet (Fig. 3E-F). Administration of sitagliptin led to an increase in amylase activity when administered to mice on both normal chow diet and high fat diet (Fig. 3E). Histopathological analysis did not indicate an increased signal for pancreatitis from drug treatment (Fig. S3). The apparent discrepancy from the four methods of assessing pancreatitis may be explained by the relative sensitivities of the methods. Histopathology is a subjective method of analysis of the pancreas and, whilst we think it is a valuable assessment tool, we wanted 1) a more quantitative way for assessing pancreatitis, and 2) more than one method to assess pancreatitis. We therefore chose to also assess pancreatitis by measuring pancreatic Reg3b content, and plasma amylase and lipase content. Reg3b is an indicator of tissue regeneration and is upregulated when pancreata are damaged e.g. by pancreatitis. Thus, a change in the content of this protein in the pancreas can be a measure for mild pancreatitis, where pancreatic tissue is available for analysis. Assessment of changes in plasma amylase and lipase in mild pancreatitis is difficult as these can be cleared by the renal system; this is problematic for early diagnosis of pancreatitis, but is the only measure available in the clinic where pancreatic material is not available for analysis.

Thus, our current data do not suggest a clinical lesion, as defined in the human setting. However, as we observed an effect of sitagliptin to increase two out of our three measures for pancreatitis, longer term studies which follow larger cohorts of mice until the end of their natural life may shed more light on the risk for pancreatitis and pancreatic cancer in this model.

## Supporting Information

Figure S1 **Mice were rendered glucose intolerant following 8 weeks on a high fat diet.** Time course (A) and area under the curve (B) of 16 week old male C57BL/6 mice on high fat diet (HFD) and normal diet (ND) after 8 weeks on differential diet. (C) Plasma was extracted from mice 1 h following intraperitoneal injection of glucose (1 g/kg) and saline or sitagliptin (10 mg/kg), and plasma GLP-1 was measured as described in [45].
(TIF)

Figure S2 **Manual verification of macro calculations.** The percentage of Reg3b positive areas out of 10 (manual; A) and 20 (macro; B) randomly chosen fields from pancreatic sections from mice treated with saline or exendin-4 were scored by visual examination. In the manual verification, a field was considered positive when there was at least one positive signal within the optical field regardless of area of signal. ND, normal chow diet; HFD, high fat diet.
(TIF)

Figure S3 **Histopathology report.** Analyses were carried out as described in ‘Materials and Methods’. Numbers in brackets indicate number of positive observations by the total number of observations made.
(TIF)
