# Supplementary figures and images for: Divergent Effects of Liraglutide, Exendin-4, and Sitagliptin on Beta-Cell Mass and Indicators of Pancreatitis in a Mouse Model of Hyperglycaemia
Source: PLoS One. 2014 Aug 13;9(8):e104873. doi: 10.1371/journal.pone.0104873 (PMC4132080; doi:10.1371/journal.pone.0104873)

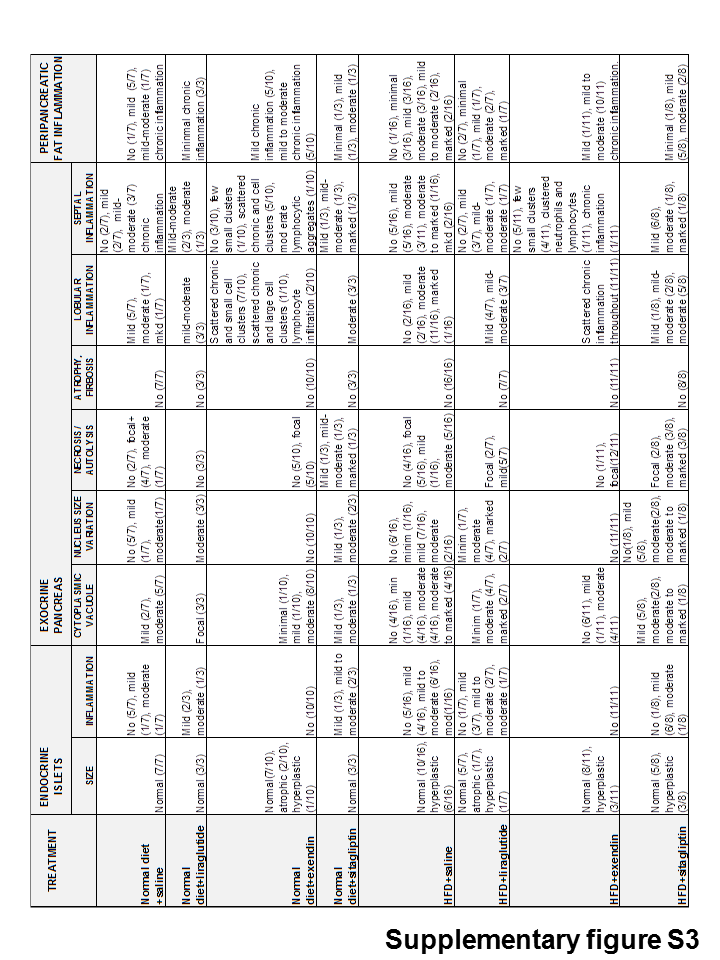

Supplement: Figure S3 — Histopathology report. Analyses were carried out as described in ‘Materials and Methods’. Numbers in brackets indicate number of positive observations by the total number of observations made. (TIF) [file pone.0104873.s003.tif]
